# Comparison of Modified Pectoral Nerve Block and Erector Spinae Plane Block for Postoperative Analgesia and Surgical Stress Response Following Modified Radical Mastectomy: A Prospective, Randomised, Blinded Study

**DOI:** 10.1155/tbj/2712790

**Published:** 2026-10-04

**Authors:** Avnika Laller, Lalit Gupta, Devang Bharti, Kapil Chaudhary

**Affiliations:** ^1^ Department of Anaesthesiology and Intensive Care Maulana Azad Medical College and Associated Hospitals New Delhi India mamc.ac.in

**Keywords:** cortisol, erector spinae plane block, IL-6, modified radical mastectomy, NLR, patient satisfaction, pectoral nerve block, postoperative analgesia, surgical stress response

## Abstract

**Background:**

Erector spinae plane block (ESPB) and modified pectoral nerve block (PECS‐II) are being increasingly used to control pain following breast surgeries. However, evidence comparing their effects on postoperative analgesia and surgical stress response is limited. The current study compared analgesic efficacy and surgical stress response attenuation of ESPB and PECS‐II in patients undergoing modified radical mastectomy (MRM).

**Methods:**

In this prospective, randomised, comparative study, 42 patients undergoing elective MRM were randomly divided to receive either ESPB or PECS‐II. Both groups received 30 mL of 0.2% ropivacaine with 0.5 μg/kg dexmedetomidine after induction of general anaesthesia (GA). The primary outcome was time to first rescue analgesia requirement. Secondary outcomes were postoperative numerical rating scale (NRS) scores, rescue analgesic consumption, intraoperative fentanyl requirement, serum cortisol, interleukin‐6 (IL‐6) and neutrophil‐to‐lymphocyte ratio (NLR), patient satisfaction and block‐related complications.

**Results:**

The mean time to first rescue analgesia was significantly larger in the PECS‐II group than in the ESPB group (9.05 ± 1.12 h vs 6.95 ± 1.16 h; *p* < 0.001). Patients receiving PECS‐II had lower 24‐h diclofenac (114.3 ± 38.4 mg vs 146.4 ± 16.4 mg; *p* = 0.002) and tramadol consumption (7.1 ± 17.9 mg vs 23.8 ± 25.6 mg; *p* = 0.021). NRS scores were lower in the PECS‐II group at 6 h postoperatively (*p* < 0.001). Intraoperative fentanyl requirement was comparable between the two groups (*p* = 0.133). Postoperative serum cortisol (363.48 ± 41.79 vs 495.95 ± 52.20 nmol/L), IL‐6 (14.48 ± 4.39 vs 24.43 ± 6.33 pg/mL) and NLR (4.15 ± 0.55 vs 4.98 ± 0.85) were also lower in the PECS‐II group (*p* ≤ 0.001). Patient satisfaction scores were higher following PECS‐II (5.62 ± 0.74 vs 5.05 ± 0.67; *p* = 0.022). No block‐related complications were observed in either group.

**Conclusion:**

PECS‐II provided longer postoperative analgesia than ESPB in patients undergoing MRM under GA. Exploratory analyses showed lower postoperative analgesic consumption, lower pain scores at 6 h, lower postoperative stress response markers and higher patient satisfaction with PECS‐II. These secondary findings should be considered hypothesis‐generating and warrant confirmation in larger, adequately powered studies. Intraoperative fentanyl requirement was comparable between the two groups.

**Trial Registration:** Clinical Trials Registry: CTRI/2024/04/066198

## 1. Introduction

Breast cancer is one of the most commonly diagnosed malignancies in women and constitutes a major public health burden, particularly in low‐ and middle‐income‐group countries [1]. Surgical management remains the cornerstone of treatment, with modified radical mastectomy (MRM) being widely performed in the majority of patients [2, 3]. Despite advances in perioperative pain management, postoperative pain remains a significant concern in patients undergoing MRM due to the involvement of extensive tissue dissection in breast and axillary regions. If not adequately controlled, this acute postoperative pain may impair recovery, increase analgesic use, prolong hospital stay and contribute to the development of persistent postmastectomy chronic pain syndromes [4, 5]. For this reason, effective perioperative analgesia has become an integral part of enhanced recovery techniques in breast surgery.

Ultrasound‐guided regional anaesthesia (RA) techniques are being increasingly adopted in perioperative multimodal analgesic strategies, because they provide excellent analgesia and consequently reduce perioperative opioid use and opioid‐related side effects [6, 7]. Among the available fascial plane blocks, erector spinae plane block (ESPB) and modified pectoral nerve block (PECS‐II) have gained considerable attention because of their relative simplicity, good safety profile and efficacy in breast surgery [8–10].

ESPB involves deposition of local anaesthetic (LA) drug in the fascial plane deep to the erector spinae muscle, to involve thoracic spinal nerves and potentially the paravertebral space [10, 11]. The PECS‐II, however, provides analgesia by targeting the pectoral nerves, intercostobrachial nerve, long thoracic nerve, thoracodorsal nerve and lateral branches of the intercostal nerves [8, 9]. Several studies have attempted to compare these blocks in patients undergoing breast surgeries. Although most of them agree that both these blocks provide effective postoperative analgesia, there is conflicting evidence regarding the superiority of one technique over the other in terms of analgesic duration, opioid requirement and patient satisfaction [12–18].

Surgical trauma and perioperative pain elicit a complex neuroendocrine and inflammatory stress response by activating the hypothalamic–pituitary–adrenal (HPA) axis and releasing inflammatory mediators [19, 20]. Biomarkers, such as serum cortisol, interleukin‐6 (IL‐6) and neutrophil‐to‐lymphocyte ratio (NLR), have been used to quantify this perioperative stress and inflammation [21–23]. RA techniques may attenuate this stress response by blocking nociceptive transmission from the surgical site. Although previous studies have compared the efficacy of ESPB and PECS‐II, relatively few have evaluated their effect on perioperative stress response markers, and the direct comparative evidence remains limited. To the best of our knowledge, no previous study has simultaneously compared complementary endocrine and inflammatory stress response markers, namely serum cortisol, IL‐6 and NLR, while evaluating the analgesic efficacy of these two RA techniques.

Therefore, this randomised controlled trial was undertaken to compare ultrasound‐guided ESPB and PECS‐II in women undergoing MRM under general anaesthesia (GA). The primary objective was to compare the time to first rescue analgesic. Secondary objectives included comparison of postoperative pain scores (numerical rating scale [NRS] scores), rescue analgesic consumption, perioperative stress markers (serum cortisol, IL‐6 and NLR), patient satisfaction (Likert scale scores) and block‐related complications.

## 2. Methods

### 2.1. Study Design and Participants

This prospective, blinded, randomised controlled trial was conducted in the Department of Anaesthesiology and Intensive Care, Maulana Azad Medical College, New Delhi, India, between May 2024 and June 2025. Ethical approval for conducting the study was granted by the Institutional Ethics Committee, Maulana Azad Medical College (F.1/IEC/MAMC/MD/MS/108/01/2024/No. 54, dated 3 April 2024). Written informed consent was obtained from all the participants before enrolment, and the principles of the Declaration of Helsinki (2013) and good clinical practice guidelines were strictly followed while conducting the trial.

Female patients aged 18–65 years with American Society of Anesthesiologists (ASA) Physical Status (PS) grades I and II, scheduled to undergo elective MRM under GA, were considered eligible for inclusion. Patients with a known allergy to the study drugs, coagulopathy, thrombocytopaenia, infection at the block site, history of neuropsychiatric disorders, body mass index ≥ 30 kg/m^2^, chronic opioid use, pregnancy or inability to comprehend the pain scoring system were excluded.

### 2.2. Randomisation and Blinding

Eligible participants were randomly allocated in a 1:1 ratio to either receive ultrasound‐guided ESPB (ESPB group) or ultrasound‐guided PECS‐II (PECS‐II group). The randomisation was performed using a computer‐generated random sequence. Group allocation was concealed using sequentially numbered, opaque, sealed, white envelopes, which were opened immediately before block administration.

The ultrasound‐guided block was performed by a dedicated anaesthesiologist who was not involved in the subsequent intraoperative management and left the operating room immediately after block administration. Intraoperative anaesthesia was subsequently conducted by a second anaesthesiologist who was not present during block performance and was therefore blinded to the group allocation. The participants, intraoperative anaesthesiologists, postoperative assessors and data analysts remained blinded to the group allocation throughout the study period.

### 2.3. Anaesthetic Management and Block Technique

All patients underwent standard preanaesthetic evaluation and were kept fasting as per ASA preoperative fasting guidelines. On arrival in the operating theatre, the patients were reassessed, standard ASA monitors were attached, and baseline parameters were recorded. An intravenous (IV) access was secured, and blood samples were taken to determine baseline serum cortisol, IL‐6 and NLR levels.

The induction, maintenance and reversal of GA were standardised across the groups as per the institutional protocol. After the induction of GA, a regional block was performed under strict asepsis by an experienced anaesthesiologist, well‐versed in both techniques.

In the ESPB group, a high‐frequency linear ultrasound transducer was used to identify the transverse process and the overlying erector spinae muscle at the T4 vertebral level. After confirming the position of the tip of the needle, 30 mL of LA solution was deposited deep to the muscle and its superior and inferior spread was visualised.

In the PECS‐II group, the pectoralis major, pectoralis minor and serratus anterior muscles were identified using a high‐frequency linear ultrasound transducer. Once all these muscles were appropriately positioned in a single plane, 10 mL of LA solution was deposited between the pectoralis major and minor muscles. The transducer was then moved towards the axilla till the serratus anterior muscle was visualised above the third and fourth ribs, and 20 mL of LA solution was deposited below the muscle.

Patients in both groups received LA solution containing 0.2% ropivacaine with 0.5 μg/kg dexmedetomidine as an adjuvant. The surgery was commenced following the administration of blocks. In case intraoperative heart rate or mean arterial pressure increased by more than 20% of the baseline for longer than 5 min, additional fentanyl boluses, 0.5 μg/kg IV, were administered and recorded. Paracetamol 1 g IV was administered to all the study patients 30 min before shifting out of the theatre.

### 2.4. Outcome Measures and Postoperative Monitoring

The primary outcome was the time to first rescue analgesic requirement, defined as the time interval between completion of block administration and first postoperative rescue analgesic administration. Secondary outcomes included postoperative pain intensity assessed using the NRS, total rescue analgesic consumption in the first 24 h postoperatively, perioperative stress response markers, patient satisfaction assessed using a Likert scale, intraoperative fentanyl requirement and block‐related complications.

All the study subjects were monitored for 24 h after completion of surgery. Postoperative pain was assessed using NRS, and scores were recorded at 30 min, 2 h, 4 h, 6 h, 8 h, 12 h and 24 h postoperatively. At any point in time if the NRS score was more than 3 or if the patient complained of pain, rescue analgesia in the form of diclofenac sodium (aqueous base) 75 mg IV was administered and recorded. Subsequent doses, if required, were administered with a minimum gap of 8 h and were also recorded. If the pain was not adequately relieved 30 min after diclofenac sodium administration, a second rescue analgesic in the form of tramadol hydrochloride 50 mg was administered IV and recorded.

To assess perioperative stress response, blood samples were again taken 6 h postoperatively and sent for analysis of serum cortisol, IL‐6 and NLR levels. Patient satisfaction was assessed at 24 h postoperatively using a verbal Likert scale. Any complication attributable to regional blocks was also recorded.

### 2.5. Sample Size

The sample size for the study was based on the primary outcome, time to first rescue analgesic. Sinha et al. [12] demonstrated a mean duration of analgesia of 7.26 ± 0.69 h for PECS‐II and 5.87 ± 1.47 h for ESPB. Taking the power of the study to be 80% and a two‐sided α to be 0.05, the sample size was calculated to be 19 participants per group. Assuming an attrition rate of 10%, 21 patients were enrolled in each group.

### 2.6. Statistical Analysis

Data were entered into Microsoft Excel Professional Plus 2021 (Microsoft Corporation, Redmond, WA, USA) and analysed using the Statistical Package for the Social Sciences (SPSS) Version 21.0 (IBM Corp., Armonk, NY, USA).

Normality of continuous variables was assessed using the Shapiro–Wilk test. Normally distributed continuous variables were expressed as mean ± standard deviation (SD) and compared using the independent samples *t*‐test. Categorical variables were expressed as frequency and percentage and were analysed using the chi‐square test or Fisher’s exact test, as appropriate. Postoperative serum cortisol concentrations were additionally analysed using analysis of covariance (ANCOVA), with preinduction serum cortisol as the covariate and study group as the fixed factor. A two‐sided *p* value < 0.05 was considered statistically significant for the primary outcome, as the study was powered only for it (time to first rescue analgesic requirement). Analyses of secondary outcomes were considered exploratory and were interpreted cautiously in view of multiple comparisons, and therefore, *p* values for secondary outcomes should be regarded as hypothesis‐generating rather than confirmatory.

## 3. Results

A total of 49 patients were assessed for eligibility, and seven did not fulfil the inclusion criteria. The remaining 42 patients were included and were randomised to receive either ESPB or PECS‐II. All the enrolled patients received the allocated intervention and were included in the final analysis (Figure 1).

**FIGURE 1:**
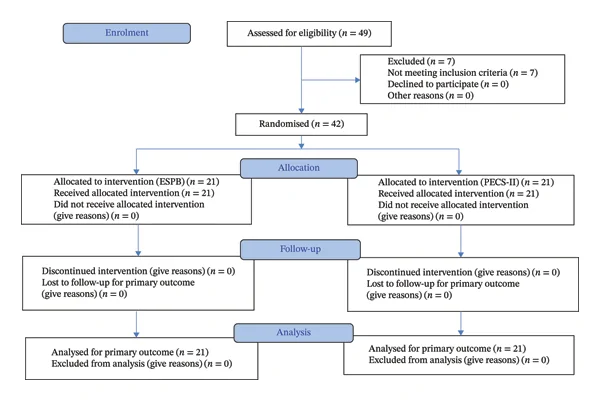
CONSORT flow diagram. *n*: number of patients.

The two groups were comparable with respect to age, weight and ASA PS, with no statistically significant difference observed between them (Table 1).

**TABLE 1:** Demographic characteristics of the study subjects.

Variable	ESPB group (*n* = 21)	PECS‐II group (*n* = 21)	*p*‐Value
Age (years) (mean ± SD)	45.43 ± 7.82	49.33 ± 8.08	0.119
Weight (kg) (mean ± SD)	57.29 ± 5.81	57.14 ± 5.46	0.935
ASA I *n* (%)	16 (76.2)	15 (71.4)	0.726
ASA II *n* (%)	5 (23.8)	6 (28.6)	

*Note:* PECS‐II: modified pectoral nerve block, *n*: number of patients.

Abbreviations: ESPB, erector spinae plane block; SD, standard deviation.

The mean time to first rescue analgesic was significantly longer in the PECS‐II group than in the ESPB group (9.05 ± 1.12 h [95% CI: 8.54–9.56] vs 6.95 ± 1.16 h [95% CI: 6.42–7.48]; *p* < 0.001) (Table 2).

**TABLE 2:** Analgesic outcomes in the study groups.

Variable	ESPB group (*n* = 21)	PECS‐II group (*n* = 21)	*p*‐Value
Time to first rescue analgesia (h) (mean ± SD)	6.95 ± 1.16 (95% CI: 6.42–7.48)	9.05 ± 1.12 (95% CI: 8.54–9.56)	< 0.001
Patients requiring intraoperative fentanyl *n* (%)	7 (33.3)	3 (14.3)	0.133
Diclofenac consumption (mg) (mean ± SD)	146.4 ± 16.4 (95% CI: 138.8–153.8)	114.3 ± 38.4 (95% CI: 96.8–131.8)	0.002
Tramadol consumption (mg) (mean ± SD)	23.8 ± 25.6 (95% CI: 12.1–35.5)	7.1 ± 17.9 (95% CI: −1.1–15.3)	0.021

*Note:* PECS‐II: modified pectoral nerve block; *n*: number of patients.

Abbreviations: 95% CI, 95% confidence interval; ESPB, erector spinae plane block; SD, standard deviation.

Postoperative NRS scores were comparable between the groups at 30 min, 2 h, 4 h, 12 h and 24 h after the surgery. However, at 6 h, NRS scores were significantly lower in the PECS‐II group (2.10 ± 0.44 vs 3.43 ± 0.68; *p* < 0.001). A statistically significant difference was again observed at 8 h postoperatively with NRS scores being lower in the ESPB group (Figure 2).

**FIGURE 2:**
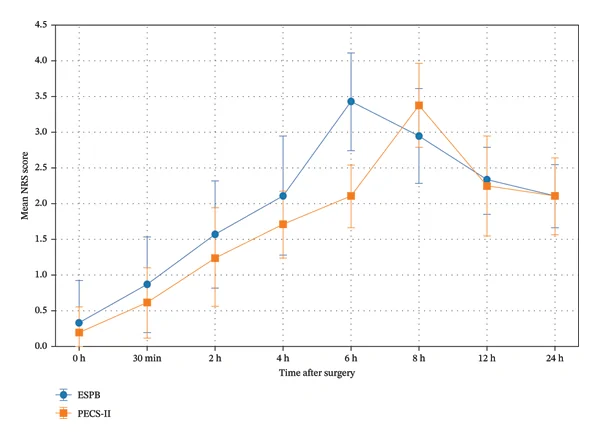
NRS scores in the study groups. NRS: numerical rating scale. Statistical significance at 6‐ and 8‐h time intervals.

The mean 24‐h diclofenac consumption was significantly lower in the PECS‐II group than in the ESPB group (114.3 ± 38.4 [95% CI: 96.8–131.8] mg vs 146.4 ± 16.4 [95% CI: 138.8–153.8] mg; *p* = 0.002) (Table 2). Similarly, the mean 24‐h tramadol consumption was also lower in the PECS‐II group (7.1 ± 17.9 [95% CI: −1.1–15.3] mg vs 23.8 ± 25.6 [95% CI: 12.1–35.5] mg; *p* = 0.021) (Table 2).

Supplemental intraoperative fentanyl was required in three patients in the PECS‐II group and seven patients in the ESPB group, with the difference not reaching statistical significance (*p* = 0.133) (Table 2).

The preoperative baseline levels of all three stress response markers were comparable between the groups. At 6 h postsurgery, concentrations of serum cortisol (363.48 ± 41.79 [95% CI: 344.5–382.5] nmol/L vs 495.95 ± 52.20 [95% CI: 472.2–519.7] nmol/L; *p* < 0.001), IL‐6 (14.48 ± 4.39 [95% CI: 12.48–16.47] pg/mL vs 24.43 ± 6.33 [95% CI: 21.55–27.31] pg/mL; *p* < 0.001) and NLR (4.15 ± 0.55 [95% CI: 3.91–4.40] vs 4.98 ± 0.85 [95% CI: 4.59–5.36]; *p* = 0.001) were significantly lower in the PECS‐II group than in the ESPB group (Table 3). After adjustment for preinduction serum cortisol using ANCOVA, the between‐group difference remained statistically significant (*p* < 0.001).

**TABLE 3:** Perioperative stress response markers in the study groups.

Variable	ESPB group (*n* = 21)	PECS‐II group (*n* = 21)	*p*‐Value
Serum cortisol (nmol/L) (mean ± SD)			
Preinduction	283.57 ± 38.24	240.57 ± 30.60	0.061
6 h postextubation	495.95 ± 52.20 (95% CI: 472.2–519.7)	363.48 ± 41.79 (95% CI: 344.5–382.5)	< 0.001^∗^
IL‐6 (pg/mL) (mean ± SD)			
Preinduction	3.81 ± 1.54	2.90 ± 0.77	0.190
6 h postextubation	24.43 ± 6.33 (95% CI: 21.55–27.31)	14.48 ± 4.39 (95% CI: 12.48–16.47)	< 0.001
NLR (mean ± SD)			
Preinduction	2.75 ± 0.49	2.59 ± 0.39	0.167
6 h postextubation	4.98 ± 0.85 (95% CI: 4.59–5.36)	4.15 ± 0.55 (95% CI: 3.91–4.40)	0.001

*Note:* PECS‐II: modified pectoral nerve block, *n*: number of patients.

Abbreviations: 95% CI, 95% confidence interval; ESPB, erector spinae plane block; SD, standard deviation.

^∗^Between‐group comparison performed using analysis of covariance (ANCOVA), with preinduction serum cortisol as the covariate and study group as the fixed factor. The adjusted between‐group difference remained statistically significant (*F* = 62.61, *p* < 0.001).

The patient satisfaction scores, assessed using a 7‐point Likert scale, were significantly higher in the PECS‐II group than in the ESPB group (5.62 ± 0.74 [95% CI: 5.28–5.96] vs 5.05 ± 0.67 [95% CI: 4.74–5.35], *p* = 0.022) (Table 4). No block‐related complications were seen in either group.

**TABLE 4:** Patient satisfaction and complications.

Variable	ESPB group (*n* = 21)	PECS‐II group (*n* = 21)	*p*‐Value
Likert satisfaction score (1–7) (mean ± SD)	5.05 ± 0.67 (95% CI: 4.74–5.35)	5.62 ± 0.74 (95% CI: 5.28–5.96)	0.022
Block‐related complications *n* (%)	0 (0)	0 (0)	—

*Note:* PECS‐II: modified pectoral nerve block, *n*: number of patients.

Abbreviations: 95% CI, 95% confidence interval; ESPB, erector spinae plane block; SD, standard deviation.

## 4. Discussion

This prospective randomised study found PECS‐II superior to ESPB for postoperative analgesia in patients undergoing MRM. Patients receiving PECS‐II experienced significantly longer duration of postoperative analgesia, lower pain scores at clinically relevant 6‐h intervals, lower rescue analgesic consumption, greater attenuation of perioperative stress response and higher patient satisfaction scores. No block‐related complication was observed in either of the groups.

MRM involves extensive dissection of the breast, pectoral region and axilla. PECS‐II directly targets medial and lateral pectoral nerves, long thoracic nerve, thoracodorsal nerve, intercostobrachial nerve and lateral branches of the intercostal nerves supplying the breast and the axilla. In contrast, ESPB relies on the indirect spread of LA within the fascial plane, with variable spread to the ventral rami and paravertebral space [11]. This direct and more specific coverage of the nerves involved in the procedure may explain the superior analgesic outcomes seen with PECS‐II.

The findings of the current study are consistent with those of Sinha et al., who reported lower postoperative morphine consumption and longer duration of analgesia in the PECS‐II group than in the ESPB group in MRM patients [12]. Similar results were obtained by Altiparmak et al., who observed significantly reduced tramadol requirement and lower pain scores following the administration of PECS‐II block in patients undergoing MRM [13]. Gad et al. [14] and Bakeer et al. [24] also found reduced opioid requirement and lower postoperative pain scores with PECS‐II. Additional studies conducted by Singariya et al. [16], Fattah et al. [18], Mohsin et al. [25] and Shanmugam et al. [26] demonstrated either lower postoperative opioid consumption, prolonged analgesia or lower pain scores with PECS‐II than ESPB, supporting the findings of the present study.

However, a minority of comparative studies favour ESPB over PECS‐II. Mahajan et al. documented a delayed requirement for the first rescue analgesic and lower visual analogue scale (VAS) scores with ESPB, concluding that ESPB provided superior postoperative analgesia than PECS‐II in MRM patients [15]. Similarly, Bhavani et al. observed a longer time to first rescue analgesia, lower NRS scores and reduced postoperative analgesic requirement with ESPB [27]. More recently, Bhattacharya et al. reported lower postoperative pain scores, lower rescue analgesic consumption and longer duration of analgesia in favour of ESPB [17]. The findings of these studies are in contrast to those observed in the current study.

Several factors may account for these conflicting observations. First, there exists a substantial difference in the timing of block administration. In some of the studies favouring ESPB, block was administered before the induction of anaesthesia with confirmation of sensory blockade, potentially allowing optimal spread of the LA [17]. Second, LA regimens differed considerably among the studies, with different agents, adjuvants and volumes used. Third, the primary outcome measures varied considerably, ranging from postoperative opioid consumption and pain scores to quality of recovery and patient satisfaction indices. Finally, the inherently variable LA spread associated with ESPB may result in substantial interstudy variability [11].

The current study found the duration of analgesia in the PECS‐II group to be around 2 hours longer than in the ESPB group. This difference resulted in significantly lower postoperative diclofenac and tramadol consumption. Intraoperative fentanyl consumption was also lower in the PECS‐II group; however, the difference did not achieve statistical significance. Collectively, these findings suggest that PECS‐II provided more effective postoperative analgesia in MRM patients. Taken together, these findings suggest that PECS‐II provided superior postoperative analgesia, whereas intraoperative fentanyl requirement was comparable between the groups.

Both PECS‐II and ESPB resulted in overall low pain scores, indicating the effectiveness of both techniques in managing postoperative pain, but patients who received PECS‐II reported significantly lower NRS scores, compared to the ESPB group, at 6 h postoperatively. Interestingly, NRS scores were lower in the ESPB group at the 8‐h interval, despite the superiority of PECS‐II in terms of duration of analgesia and analgesic consumption. This isolated finding should be interpreted with caution. Because rescue analgesia was administered according to patient requirement, pain scores recorded after rescue analgesic administration may not accurately reflect the intrinsic analgesic efficacy of the regional block. Therefore, the 8‐h pain scores should be interpreted in the context of this potential confounding effect.

One of the major strengths of this study is the simultaneous assessment of multiple biochemical markers of the surgical stress response. To the best of our knowledge, few previous studies have simultaneously compared endocrine and inflammatory stress response markers in patients receiving ESPB and PECS‐II. In the present study, postoperative serum cortisol, IL‐6 and NLR were significantly lower in the PECS‐II group, despite no significant difference in intraoperative fentanyl requirement. This apparent discordance may be explained by the fact that intraoperative fentanyl administration in our study was protocolised and triggered by sustained haemodynamic changes, whereas serum cortisol, IL‐6 and NLR, measured 6 h postoperatively, reflect endocrine and inflammatory responses to the surgical insult over a different time course. Importantly, a similar pattern has been reported by Gad et al., who found comparable intraoperative fentanyl requirements but lower postoperative serum cortisol levels with PECS‐II than with ESPB in patients undergoing MRM [14]. Studies evaluating conventional haemodynamic‐guided intraoperative opioid administration have also highlighted that haemodynamic responses may not fully capture the complexity of perioperative nociception and surgical stress response and may not necessarily correspond to subsequent biochemical stress responses [28, 29]. In our study, the difference in postoperative serum cortisol remained statistically significant after adjustment for preinduction cortisol using ANCOVA, strengthening the observed between‐group association. Cortisol reflects activation of the HPA axis, and IL‐6 and NLR are markers of systemic inflammation. Simultaneous reduction in all three markers suggests a potential attenuation of both endocrine and inflammatory components of the surgical stress response with PECS‐II. However, these secondary findings were exploratory and should be interpreted as hypothesis‐generating rather than confirmatory.

Patient satisfaction scores were significantly higher in the PECS‐II group, likely reflecting the effect of prolonged analgesia and reduced requirement of postoperative analgesics. Importantly, no block‐related complication was observed in either of the groups, emphasising the safety of both the techniques under ultrasound guidance.

Certain limitations of this study need to be acknowledged. It was a single‐centre study with a relatively small sample size, limiting the generalisability of the results. The study was powered only for the primary outcome, and analyses of secondary outcomes were exploratory; therefore, these findings should be interpreted as hypothesis‐generating and require confirmation in larger adequately powered studies. Postoperative pain scores obtained after administration of rescue analgesia may have been influenced by the analgesic intervention itself, limiting their ability to reflect the intrinsic efficacy of the study blocks at subsequent assessment time points. Follow‐up was limited to the first 24 h in the postoperative period, and chronic pain associated with mastectomy was not evaluated. In addition, as the block was administered postinduction of anaesthesia, objective assessment of the extent of sensory blockade could not be performed.

## 5. Conclusion

The findings of the present study demonstrate that ultrasound‐guided PECS‐II provided longer postoperative analgesia than ESPB in patients undergoing MRM under GA. PECS‐II was also associated with lower postoperative analgesic consumption, pain scores and biochemical stress response markers, as well as higher patient satisfaction. However, these secondary findings were exploratory and should be interpreted as hypothesis‐generating. Intraoperative fentanyl requirements were comparable between the two techniques. Both blocks demonstrated a favourable safety profile. Overall, these findings suggest that PECS‐II may offer advantages over ESPB for postoperative analgesia, with a potential additional effect on the postoperative stress response, which warrants confirmation in larger, adequately powered studies.

## Author Contributions

Avnika Laller: data collection, investigation, formal analysis and writing–original draft. Lalit Gupta: conceptualisation, methodology, visualisation, validation, supervision, project administration and writing–review and editing. Devang Bharti: formal analysis, writing–original draft, validation, data curation and writing–review and editing. Kapil Chaudhary: methodology, software, resources, supervision and writing–review and editing.

## Funding

No funding was obtained for this research. The open access fees for this article is fully funded by the One Nation One Subscription (ONOS) initiative of Government of India.

## Disclosure

All authors read and approved the final manuscript.

## Ethics Statement

The study was approved by the Institutional Ethics Committee, Maulana Azad Medical College and Associated Hospitals, New Delhi, India (Approval No. F.1/IEC/MAMC/MD/MS/108/01/2024/No. 54, dated 3 April 2024). Written informed consent was obtained from all participants before enrolment.

## Consent

Please see the Ethics Statement.

## Conflicts of Interest

The authors declare no conflicts of interest.

## Data Availability

The data that support the findings of this study are available from the corresponding author upon reasonable request.
